# Supplementary material for: Intrinsic signaling pathways modulate targeted protein degradation
Source: Nat Commun. 2024 Jul 2;15:5379. doi: 10.1038/s41467-024-49519-z (PMC11220168; doi:10.1038/s41467-024-49519-z)
Supplement: Supplementary file 1 — Supplementary Information [file 41467_2024_49519_MOESM1_ESM.pdf]

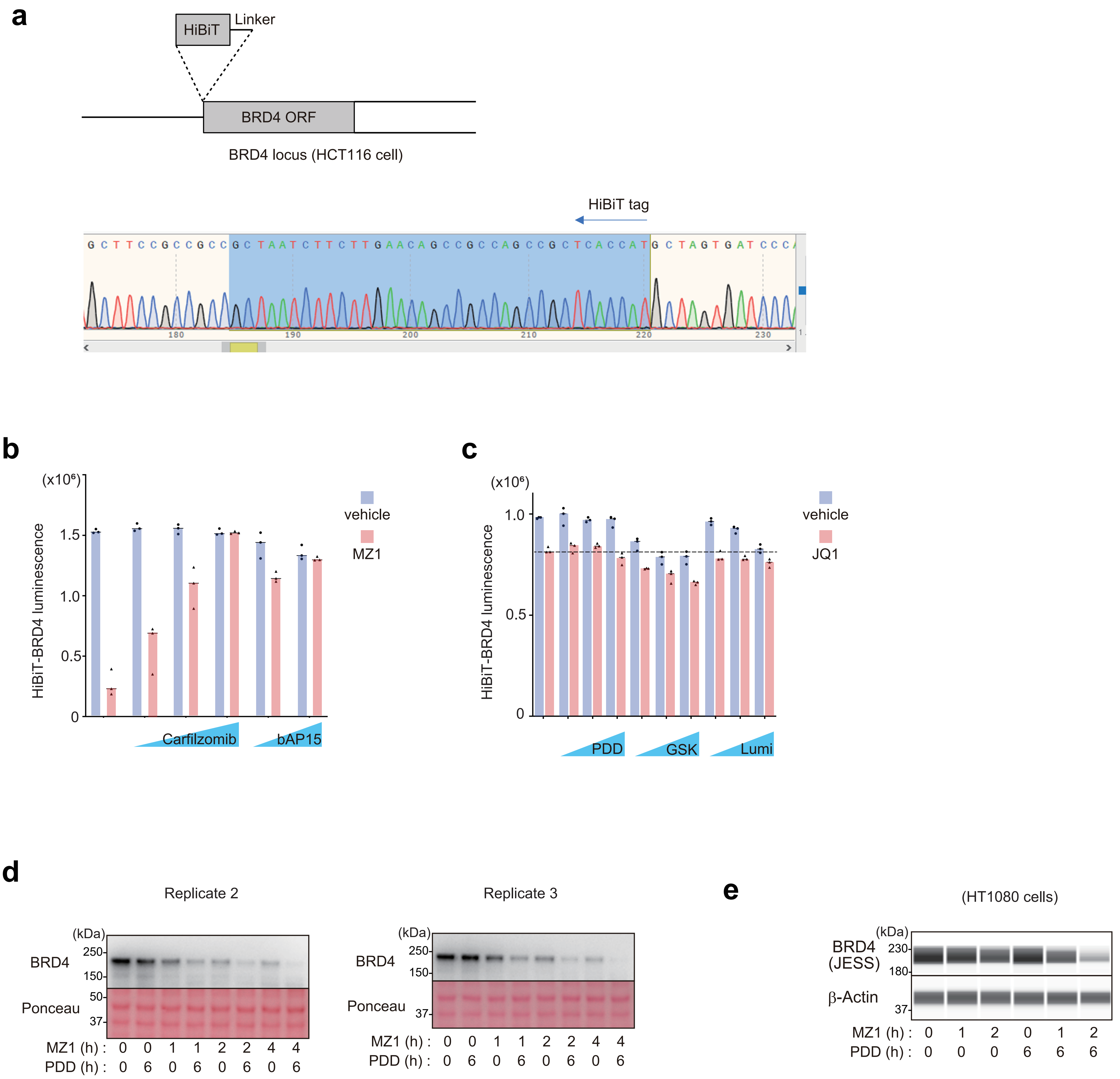

Supplementary Figure 1

a, Scheme of HiBiT-tagged BRD4 knock-in HCT116 cells.

b, HiBiT-BRD4 cells were treated with either DMSO, carfilzomib (0.1, 0.3, and 1 μM), or bAP15 (1 and 3 μM) for 30 min and then treated with 100 nM MZ1 for an additional 2 h. HiBiT luminescence was measured (n = 3, biological replicates).

c, HiBiT-BRD4 cells were treated with either DMSO or 1, 3, or 10 μM inhibitors for 4 h and then treated with 100 nM JQ1 for 2 h (n = 3, biological replicates).

d, Related to Fig. 2a; the other two biological replicates are shown.

e, HT1080 cells were treated with 3 μM PDD (6 h) and/or 100 nM MZ1 (1 or 2 h), as indicated.

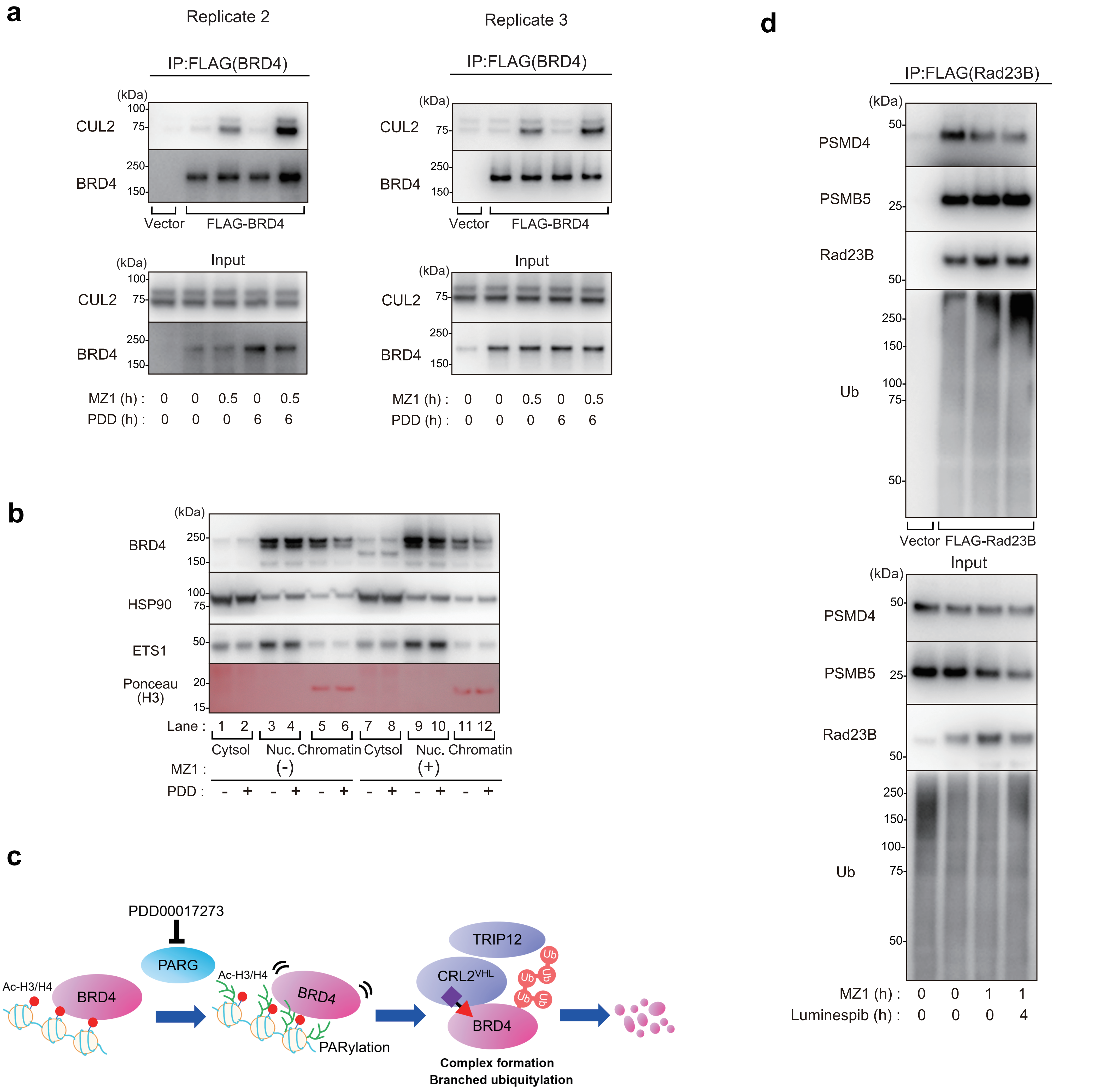

Supplementary Figure 2

a, Related to Fig. 3g–h; the other two biological replicates are shown.

b, HCT116 cells were treated with PDD (5 μM, 4 h), and cell fractionation was performed. HSP90, ETS1, and histone H3 are presented as controls for cytosolic, soluble nuclear, and chromatin fractions, respectively.

c, Schematic model.

d, 293T cells were transfected with FLAG-Rad23B (lanes 2–4), and cell lysates were subjected to immunoprecipitation using anti-FLAG antibody.

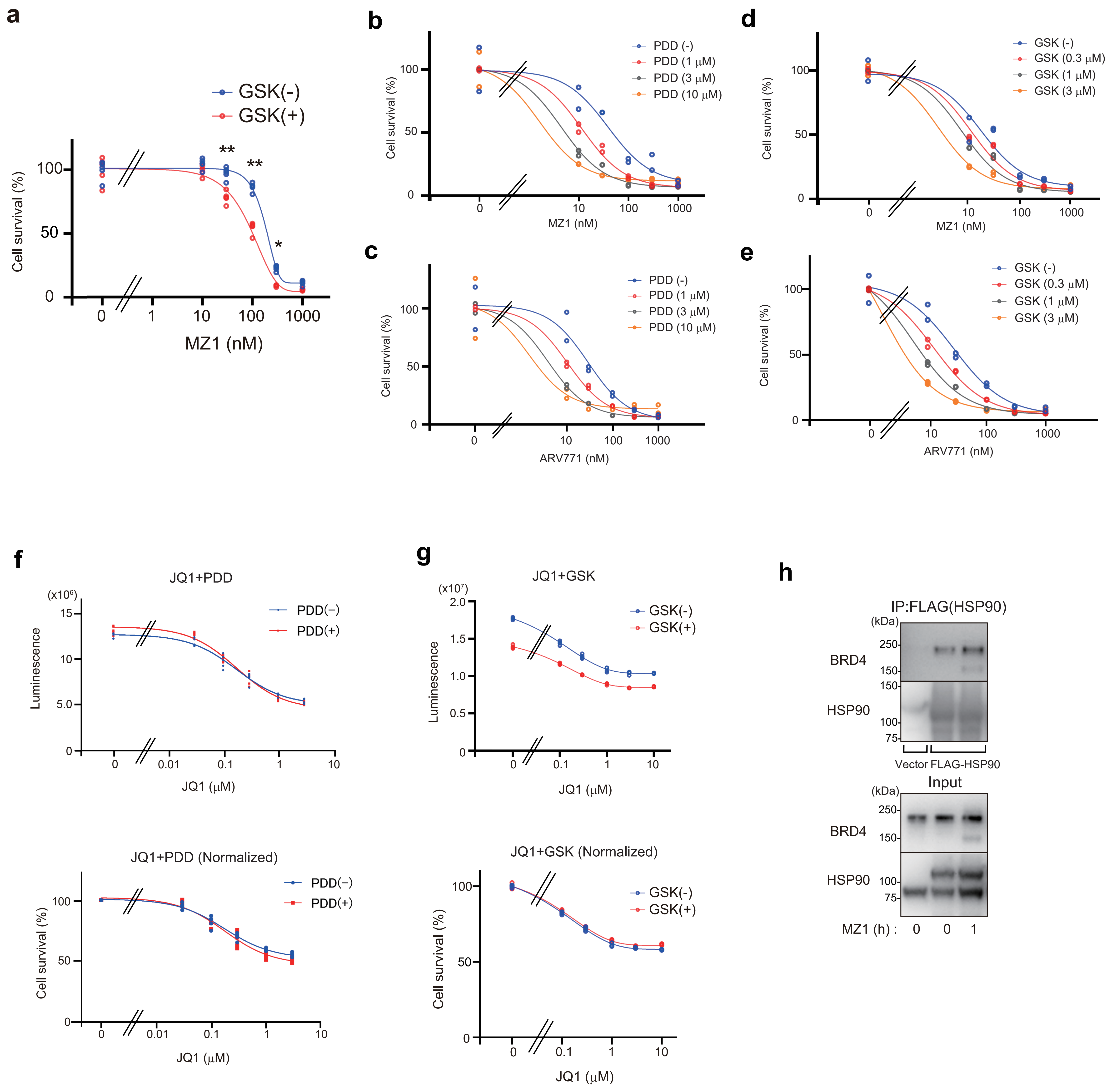

Supplementary Figure 3

a, Related to Fig. 5b(ii); the percentage of cell viability changes compared with 0 nM MZ1 is shown. Asterisk: * P = 0.003 or ** P < 0.0001 in ANOVA (n = 5, biological replicates).

b–e, Related to Fig. 5a-b, mouse B16 cells were treated with PDD or GSK together with the indicated concentration of MZ1 or ARV771 for 3 days, and cell viability was measured. (n = 2, biological replicates).

f–g, Related to Fig. 5b; PDD or GSK did not promote the cell death induced by JQ1. HeLa cells were treated either with vehicle, PDD (3 μM), or GSK (2 μM), together with the indicated concentration of JQ1 for 3 days, and cell viability was measured. In the lower panel, the percentage of cell viability changes compared with 0 nM JQ1 is shown.

h, BRD4 interacts with HSP90 in cells at the steady state. 293T cells were transfected with FLAG-HSP90 (lanes 2–3), and cell lysates were subjected to immunoprecipitation using anti-FLAG antibody.
