# Supplementary material for: Intrinsic signaling pathways modulate targeted protein degradation
Source: Nat Commun. 2024 Jul 2;15:5379. doi: 10.1038/s41467-024-49519-z (PMC11220168; doi:10.1038/s41467-024-49519-z)
Supplement: Supplementary file 2 — Reporting Summary [file 41467_2024_49519_MOESM2_ESM.pdf]

nature portfolio

Corresponding author(s): Fumiaki Ohtake

Last updated by author(s): 5/23/2024

Reporting Summary

Nature Portfolio wishes to improve the reproducibility of the work that we publish. This form provides structure for consistency and transparency in reporting. For further information on Nature Portfolio policies, see our Editorial Policies and the Editorial Policy Checklist.

Statistics

For all statistical analyses, confirm that the following items are present in the figure legend, table legend, main text, or Methods section.

n/a	Confirmed	
☐	☒	The exact sample size (*n*) for each experimental group/condition, given as a discrete number and unit of measurement
☐	☒	A statement on whether measurements were taken from distinct samples or whether the same sample was measured repeatedly
☐	☒	The statistical test(s) used AND whether they are one- or two-sided<br>*Only common tests should be described solely by name; describe more complex techniques in the Methods section.*
☒	☐	A description of all covariates tested
☐	☒	A description of any assumptions or corrections, such as tests of normality and adjustment for multiple comparisons
☐	☒	A full description of the statistical parameters including central tendency (e.g. means) or other basic estimates (e.g. regression coefficient) AND variation (e.g. standard deviation) or associated estimates of uncertainty (e.g. confidence intervals)
☐	☒	For null hypothesis testing, the test statistic (e.g. *F*, *t*, *r*) with confidence intervals, effect sizes, degrees of freedom and *P* value noted<br>*Give P values as exact values whenever suitable.*
☒	☐	For Bayesian analysis, information on the choice of priors and Markov chain Monte Carlo settings
☒	☐	For hierarchical and complex designs, identification of the appropriate level for tests and full reporting of outcomes
☒	☐	Estimates of effect sizes (e.g. Cohen's *d*, Pearson's *r*), indicating how they were calculated

Our web collection on statistics for biologists contains articles on many of the points above.

Software and code

Policy information about availability of computer code

Data collection	MS data were acquired using Xcalibur software 2.2 (Thermo Fisher Scientific). Quantitative PCR was performed using StepOne Plus RealTime PCR System (Applied biosystems). Western blot data were acquired using FUSION-FX7. EDGE V 0.70 (Vilber-Lourmat) or JESS (ProteinSimple).
Data analysis	All statistical analyses were performed using GraphPad Prism 7 (GraphPad Software). MS data were analyzed using Proteome Discoverer 2.2 or 2.4 (Thermo Fisher Scientific). Ub-AQUA/PRM analyses were performed using PinPoint software 1.3 (Thermo Fisher Scientific). Quantitative PCR data were analyzed using StepOne Software V2.2.2 (Applied biosystems).

For manuscripts utilizing custom algorithms or software that are central to the research but not yet described in published literature, software must be made available to editors and reviewers. We strongly encourage code deposition in a community repository (e.g. GitHub). See the Nature Portfolio guidelines for submitting code & software for further information.

Data

Policy information about availability of data

All manuscripts must include a data availability statement. This statement should provide the following information, where applicable:
- Accession codes, unique identifiers, or web links for publicly available datasets
- A description of any restrictions on data availability
- For clinical datasets or third party data, please ensure that the statement adheres to our policy

The raw datasets for RNA-sequencing analyses have been deposited to the GEO with the accession number GSE243615. The raw datasets for ChIP-sequencing analyses have been deposited to the GEO, with accession number GSE262938. The remaining data are available within the Article, Supplementary Information or

Source data file. Source data are provided with this paper.

Research involving human participants, their data, or biological material

Policy information about studies with human participants or human data. See also policy information about sex, gender (identity/presentation), and sexual orientation and race, ethnicity and racism.

Reporting on sex and gender	N/A
Reporting on race, ethnicity, or other socially relevant groupings	N/A
Population characteristics	N/A
Recruitment	N/A
Ethics oversight	N/A

Note that full information on the approval of the study protocol must also be provided in the manuscript.

Field-specific reporting

Please select the one below that is the best fit for your research. If you are not sure, read the appropriate sections before making your selection.

☒ Life sciences ☐ Behavioural & social sciences ☐ Ecological, evolutionary & environmental sciences

For a reference copy of the document with all sections, see nature.com/documents/nr-reporting-summary-flat.pdf

Life sciences study design

All studies must disclose on these points even when the disclosure is negative.

Sample size	Sample size estimates has been performed on previous experience to obtain reproducibility. For in vitro experiments such as Western blot and qPCR, at least two independent experiments were performed to obtain reproducible results in the analyzed biological products. We confirmed all the presented data are reproducible from repeated experiments. All sample sizes are indicated in the methods section or the figure legends.
Data exclusions	No data exclusions.
Replication	We have reproduced all the presented results in at least two independent experiments (biological replicates), and obtained similar results.
Randomization	For randomization, cell dishes were randomly chosen before treatments.
Blinding	Mass spectrometry experiments were performed blinded when appropriate. For other experiments, blinding was not relevant as all biological samples were equally analyzed in each experiment.

Reporting for specific materials, systems and methods

We require information from authors about some types of materials, experimental systems and methods used in many studies. Here, indicate whether each material, system or method listed is relevant to your study. If you are not sure if a list item applies to your research, read the appropriate section before selecting a response.

Materials & experimental systems

n/a	Involved in the study	
☐	☒	Antibodies
☐	☒	Eukaryotic cell lines
☒	☐	Palaeontology and archaeology
☒	☐	Animals and other organisms
☒	☐	Clinical data
☒	☐	Dual use research of concern
☒	☐	Plants

Methods

n/a	Involved in the study	
☐	☒	ChIP-seq
☒	☐	Flow cytometry
☒	☐	MRI-based neuroimaging

Antibodies

Antibodies used	The following antibodies were used for western blotting: anti-BRD4 (1:1000, Cell Signaling Technology, #13440, clone E2A7X), anti-

BRD2 (1:1000, Cell Signaling Technology, #5848, clone D89B4), anti-BRD3 (1:200, Santa Cruz Biotechnology, sc-515666, clone B-12), anti-PAR (1:1000, Cell Signaling Technology, #83732, clone E6F6A), anti-TRIP12 (1:1000, Proteintech, #25303-1-AP), anti-CUL2 (1:500, Santa Cruz Biotechnology, sc-166506, clone C-4), anti-VHL (1:1000, Novus Biologicals, #091504), anti-b-Actin (1:500, Santa Cruz Biotechnology, sc-47778, clone C4), anti-PARG (1:1000, Cell Signaling Technology, #66564, clone D4E6X), anti-ER-alpha (1:1000, Santa Cruz Biotechnology, sc-543, clone HC-20), anti-MEK1 (1:1000, Cell Signaling Technology, #9124), anti-MEK2 (1:1000, Cell Signaling Technology, #9125), anti-Ub (1:1000, Santa Cruz Biotechnology, sc-8017, clone P4D1), anti-HSP90 (1:500, Santa Cruz Biotechnology, sc-69703, clone 4F10), anti-ETS1 (1:1000, Cell Signaling Technology, #14069, clone D8O8A), anti-PERK (1:1000, Cell Signaling Technology, #3192, clone C33E10), and anti-Cleaved PARP1 (1:1000, Cell Signaling Technology, #5625, clone Asp214, D64E10), anti-CDK9(1:1000, Cell Signaling Technology, #2316, clone C12F7), anti-c-MET(1:1000, Cell Signaling Technology, #4560), anti-PSMD4(1:1000, Cell Signaling Technology, #3846), anti-PSMB5(1:1000, Cell Signaling Technology, #12919, clone D1H6B), anti-Rad23B(1:1000, Cell Signaling Technology, #13525, clone D4W7F). For immunoprecipitation, the anti-FLAG (20 uL agarose/sample, Sigma-Aldrich, #A2220, clone M2) or anti-BRD4 (1 ug/sample, Abcam, #ab128874)antibody was used.
For Chromatin IP-seq (ChIP-seq), anti-BRD4 antibody (Cell Signaling Technology, #13440, clone E2A7X, rabbit monoclonal, 10 μL) and Spike-in antibody (Active Motif, #61686, 2 μL) were used.

Validation

anti-BRD4 (1:1000, Cell Signaling Technology, #13440, clone E2A7X), anti-BRD2 (1:1000, Cell Signaling Technology, #5848, clone D89B4), anti-PAR (1:1000, Cell Signaling Technology, #83732, clone E6F6A), anti-PARG (1:1000, Cell Signaling Technology, #66564, clone D4E6X), anti-MEK1 (1:1000, Cell Signaling Technology, #9124), anti-MEK2 (1:1000, Cell Signaling Technology, #9125), anti-ETS1 (Cell Signaling Technology, #14069), anti-PERK (Cell Signaling Technology, #3192), and anti-Cleaved PARP1 (Cell Signaling Technology, #5625), anti-ETS1 (1:1000, Cell Signaling Technology, #14069, clone D8O8A), anti-PERK (1:1000, Cell Signaling Technology, #3192, clone C33E10), and anti-Cleaved PARP1 (1:1000, Cell Signaling Technology, #5625, clone Asp214, D64E10), anti-CDK9(1:1000, Cell Signaling Technology, #2316, clone C12F7), anti-c-MET(1:1000, Cell Signaling Technology, #4560), anti-PSMD4(1:1000, Cell Signaling Technology, #3846), anti-PSMB5(1:1000, Cell Signaling Technology, #12919, clone D1H6B), anti-Rad23B(1:1000, Cell Signaling Technology, #13525, clone D4W7F) were validated by western blotting of human cell lines in the manufacturer's web site (https://www.cellsignal.com/).

anti-BRD3 (1:200, Santa Cruz Biotechnology, sc-515666, clone B-12), anti-CUL2 (1:500, Santa Cruz Biotechnology, sc-166506, clone C-4), anti-b-Actin (1:500, Santa Cruz Biotechnology, sc-47778, clone C4), anti-ER-alpha (1:1000, Santa Cruz Biotechnology, sc-543, clone HC-20), anti-Ub (1:1000, Santa Cruz Biotechnology, sc-8017, clone P4D1), anti-HSP90 (1:500, Santa Cruz Biotechnology, sc-69703, clone 4F10) were validated by western blotting of human cell lines in the manufacturer's web site (https://www.scbt.com/ja/home).

Anti-FLAG (Sigma-Aldrich, #A2220, clone M2) was validated in the manufacturer’s web site (https://www.sigmaaldrich.com/JP/ja).

anti-TRIP12 (Proteintech, #25303-1-AP) was validated by western blotting of human cell lines in the manufacturer's web site (https://www.ptglab.co.jp/).

anti-VHL (Novus Biologicals, #091504) was validated by western blotting of human cell lines in the manufacturer's web site (https://www.novusbio.com/japan).

anti-BRD4 (Cell Signaling Technology, #13440) was validated by ChIP-seq of human chromatin in the manufacture's web site (https://www.cellsignal.jp/products/primary-antibodies/brd4-e2a7x-rabbit-mab/13440).

SSpile-in antibody was validated by ChIP-seq in the manufacture's web site (https://www.activemotif.jp/catalog/1091/chip-normalization).

Eukaryotic cell lines

Policy information about cell lines and Sex and Gender in Research

Cell line source(s)	Human HeLa, MCF7, HEK293T, and HCT116 cells were obtained from ATCC.
Authentication	Cell line authentication was not performed.
Mycoplasma contamination	All cell lines used were periodically checked by fluorescent microscopy, and no contamination was found.
Commonly misidentified lines (See ICLAC register)	No commonly misidentified cell lines were used in this study.

Plants

Seed stocks	*Report on the source of all seed stocks or other plant material used. If applicable, state the seed stock centre and catalogue number. If plant specimens were collected from the field, describe the collection location, date and sampling procedures.*
Novel plant genotypes	*Describe the methods by which all novel plant genotypes were produced. This includes those generated by transgenic approaches, gene editing, chemical/radiation-based mutagenesis and hybridization. For transgenic lines, describe the transformation method, the number of independent lines analyzed and the generation upon which experiments were performed. For gene-edited lines, describe the editor used, the endogenous sequence targeted for editing, the targeting guide RNA sequence (if applicable) and how the editor was applied.*

Authentication

Describe any authentication procedures for each seed stock used or novel genotype generated. Describe any experiments used to assess the effect of a mutation and, where applicable, how potential secondary effects (e.g. second site T-DNA insertions, mosiacism, off-target gene editing) were examined.

ChIP-seq

Data deposition

☒ Confirm that both raw and final processed data have been deposited in a public database such as GEO.

☒ Confirm that you have deposited or provided access to graph files (e.g. BED files) for the called peaks.

Data access links
May remain private before publication.

All high-throughput sequencing data generated in this study are accessible at GEO via GEO accession number GSE262938 (https://www.ncbi.nlm.nih.gov/geo/query/acc.cgi?acc=GSE262938).

Files in database submission

Cont_In_1.fastq_36.gz
Cont_In_2.fastq_36.gz
In_P_R1_36.fastq.gz
In_P_R2_36.fastq.gz
In_M60_R1_36.fastq.gz
In_M60_R2_36.fastq.gz
All_Cont_BRD4_1.fastq.gz_36.gz
All_Cont_BRD4_2.fastq.gz_36.gz
BRD4_P_R1_36.fastq.gz
BRD4_P_R2_36.fastq.gz
BRD4_M60_R1_36.fastq.gz
BRD4_M60_R2_36.fastq.gz
BRD4_Ctrl_bowtie2_hg38_SpikeInP_macs2_1e4_control_lambda.bdg
BRD4_PDD_bowtie2_hg38_SpikeInP_macs2_1e4_control_lambda.bdg
BRD4_M60_bowtie2_hg38_SpikeInP_macs2_1e4_control_lambda.bdg
BRD4_Ctrl_bowtie2_hg38_SpikeInP_macs2_1e4_treat_pileup.bdg
BRD4_PDD_bowtie2_hg38_SpikeInP_macs2_1e4_treat_pileup.bdg
BRD4_M60_bowtie2_hg38_SpikeInP_macs2_1e4_treat_pileup.bdg
BRD4_Ctrl_bowtie2_hg38_SpikeInP_macs2_1e4_peaks.narrowPeak
BRD4_PDD_bowtie2_hg38_SpikeInP_macs2_1e4_peaks.narrowPeak
BRD4_M60_bowtie2_hg38_SpikeInP_macs2_1e4_peaks.narrowPeak

Genome browser session
(e.g. UCSC)

Available at GEO

Methodology

Replicates

Single

Sequencing depth

Name: Total_reads, Unique_reads, Reads_length, Reads_type
BRD4_Ctrl_Input: 1137112858, 131395006, 36, PE
BRD4_Ctrl_BRD4: 198967904, 152718675, 36, PE
BRD4_PDD_Input: 78145281, 76366902, 36, PE
BRD4_PDD_BRD4: 54804000, 53732381, 36, PE
BRD4_M60_Input: 73075361, 71401654, 36, PE
BRD4_M60_BRD4: 58065279, 57017528, 36, PE

Antibodies

BRD4 antibody (Cell Signaling Technology, #13440, clone E2A7X, rabbit monoclonal)
Spike-in antibody (Active Motif, #61686)

Peak calling parameters

Macs2: -f --SPMR -p 1e4

Data quality

deepTools plotFingerprint, SSP

Software

fastp (version 0.23.4)
Bowtie 2 (version 2.5.1)
SAMtools (version 1.18)
MACS2 (version 2.2.9.1)
deeptools (version 3.5.5)
