# Supplementary material for: Intrinsic signaling pathways modulate targeted protein degradation
Source: Nat Commun. 2024 Jul 2;15:5379. doi: 10.1038/s41467-024-49519-z (PMC11220168; doi:10.1038/s41467-024-49519-z)
Supplement: Supplementary file 4 — Description of Additional Supplementary Files [file 41467_2024_49519_MOESM4_ESM.pdf]

Supplementary Data 1.

Related to Fig. 1e, the list of compounds used in the screening.

Supplementary Data 2.

Related to Fig. 2c, the list of genes up- or down-regulated by PDD treatment for 6h.

Supplementary Data 3.

Related to Fig. 5f, the list of genes up- or down-regulated by PDD or GSK treatment for 24h.
