# Supplementary material for: Intrinsic signaling pathways modulate targeted protein degradation
Source: Nat Commun. 2024 Jul 2;15:5379. doi: 10.1038/s41467-024-49519-z (PMC11220168; doi:10.1038/s41467-024-49519-z)

Fig. 2a

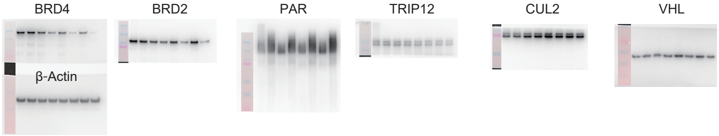

Fig. 2d

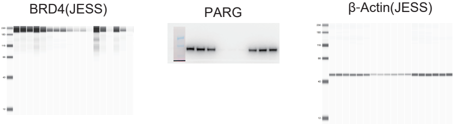

Fig. 2e

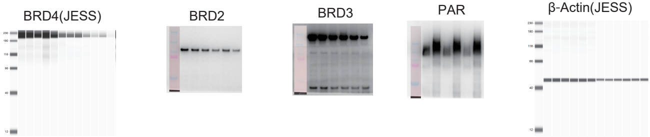

Fig. 2f

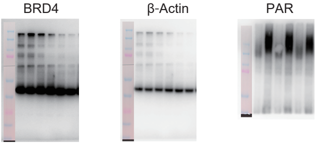

Fig. 2g

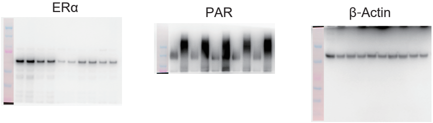

Fig. 2h

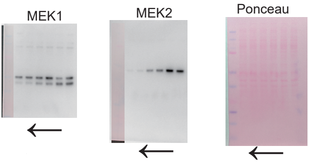

Fig.3a

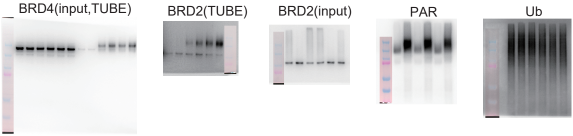

Fig.3b

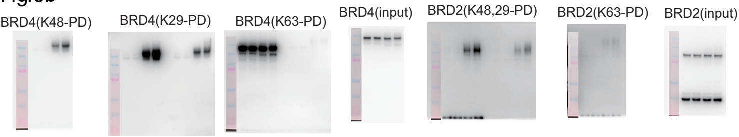

Fig.3e

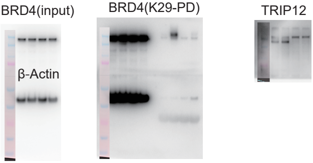

Fig.3f

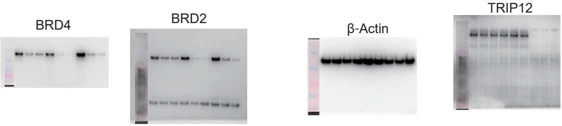

Fig.3g

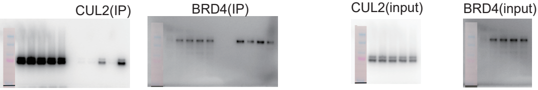

Fig.3m

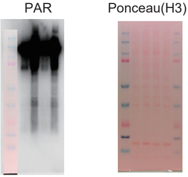

Fig. 4a

BRD4 | BRD2 | BRD3 | β-actin

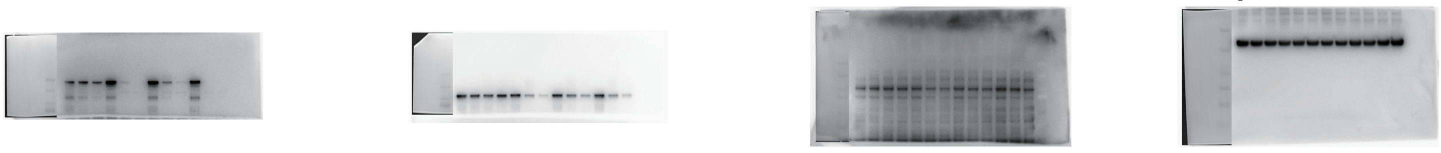

Fig. 4b

BRD4 (JESS) | Ponceau

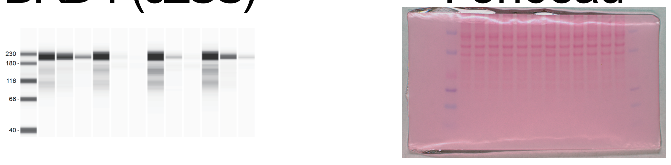

Fig. 4c

BRD4 | Ponceau

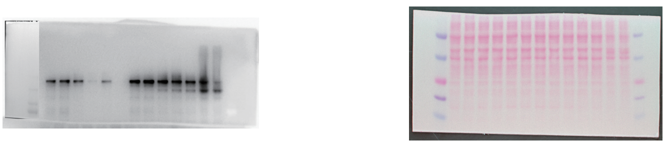

Fig. 4d

BRD4 | BRD2 | BRD3 | Ponceau

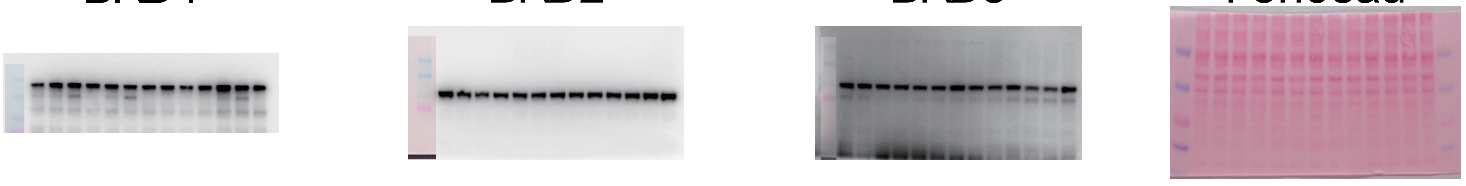

Fig. 4e

BRD4 | PERK | β-actin

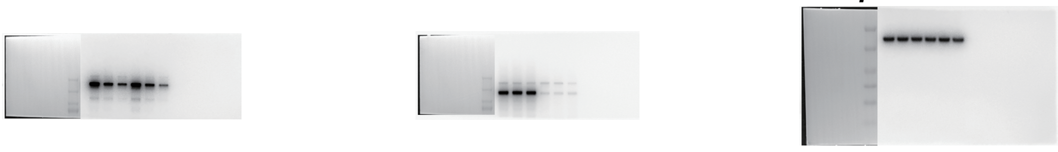

Fig. 4f

BRD4 | HSP90 | β-Actin

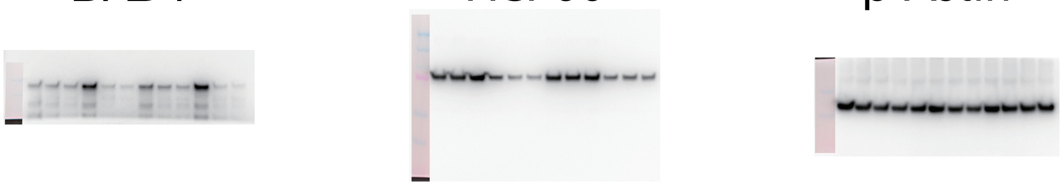

Fig. 4g

BRD4 (Input, TUBE) | BRD4 higher intensity (Input, TUBE) | Ub (Input)

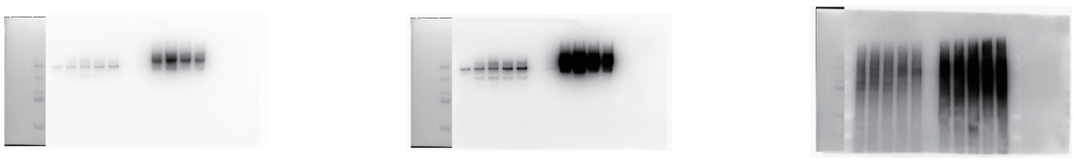

BRD4 (Input, K48-PD) | BRD4 (K63-PD,K29-PD) | BRD4 higher intensity (K63-PD , K29-PD)

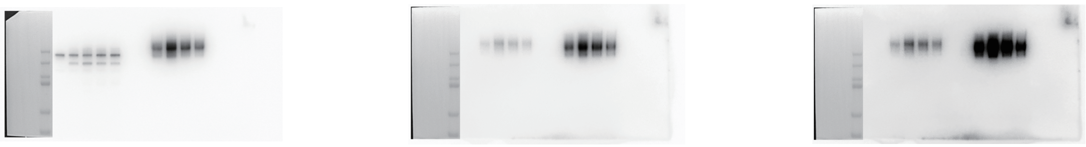

Fig. 4h

BRD2 (TUBE) | BRD2 (Input)

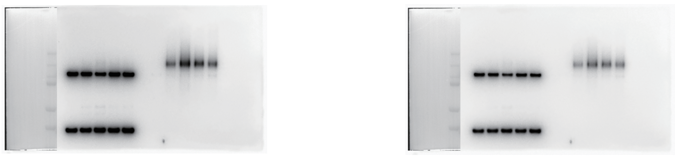

BRD2 (Input, K48-PD) | BRD2 (K63-PD,K29-PD) | BRD2 higher intensity (K63-PD , K29-PD)

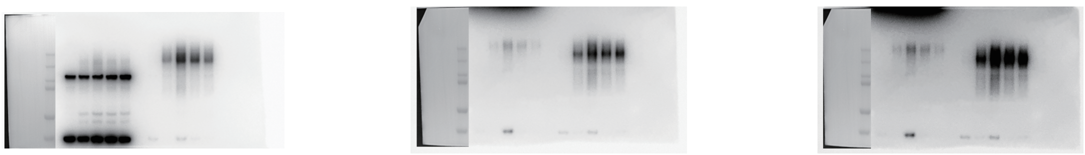

Fig. 4i

CUL2 | BRD4 (input) | BRD4 (IP)

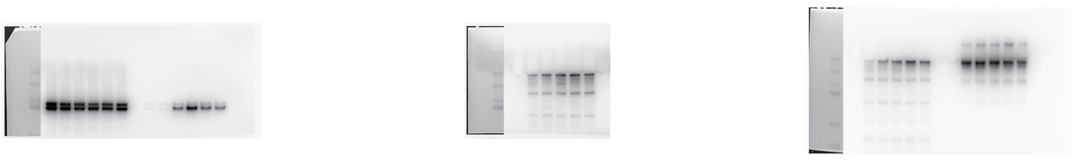

Fig. 5c

Cleaved-PARP1

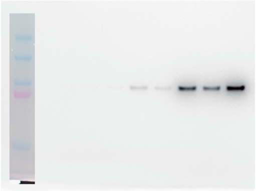

BRD4

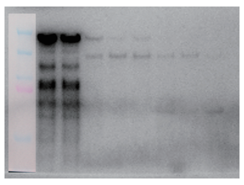

BRD2

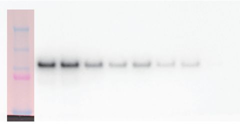

BRD3

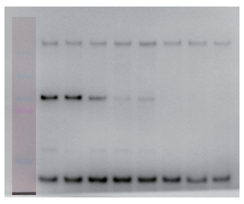

β-Actin

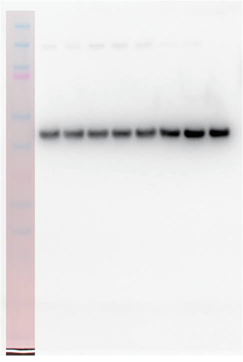

Fig. 5d

Cleaved PARP1

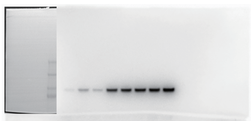

BRD4 (Light exposure)

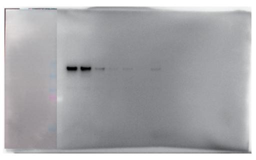

BRD4 (Dark exposure)

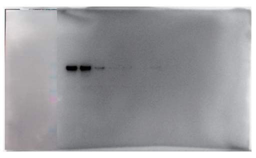

BRD2

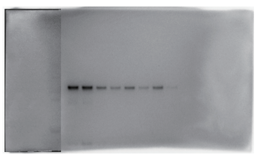

BRD3

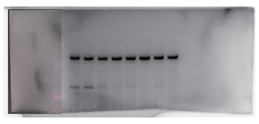

β-actin

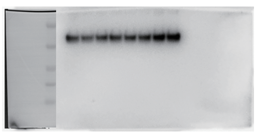

Fig. 6a BRD4 BRD2 BRD3 PAR Ponceau

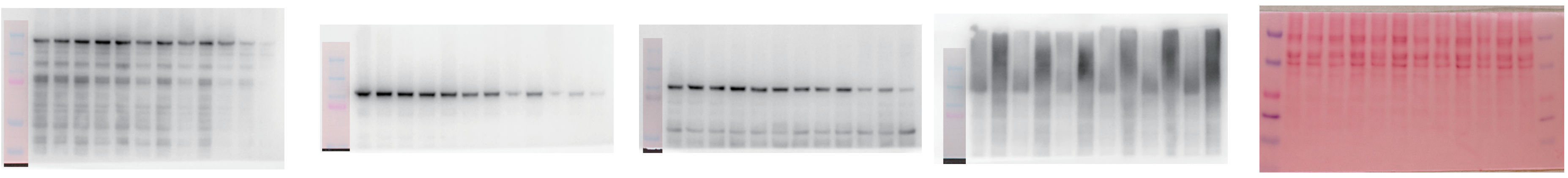

Fig. 6b BRD4 BRD2 Ponceau

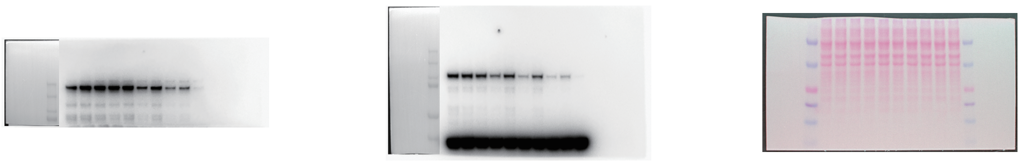

Fig. 6f CDK9 PAR β-Actin

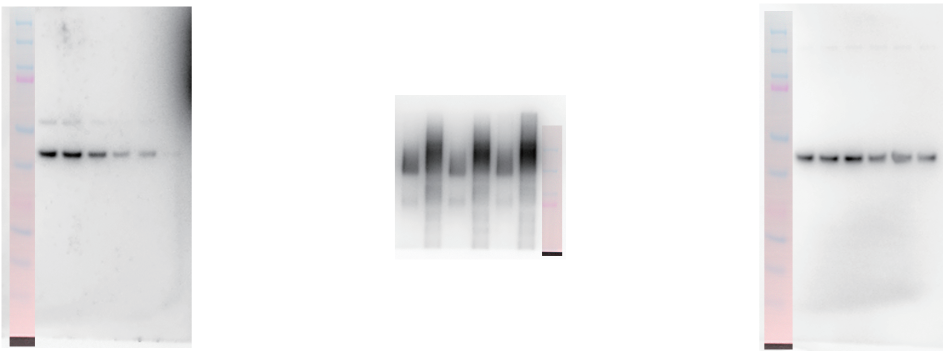

Fig. 6h CDK9 Ponceau

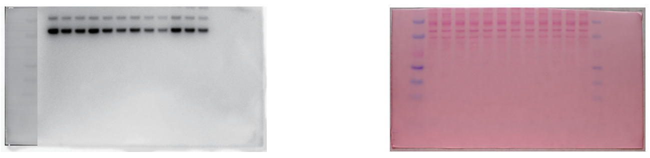

Fig. 6i ERα Ponceau

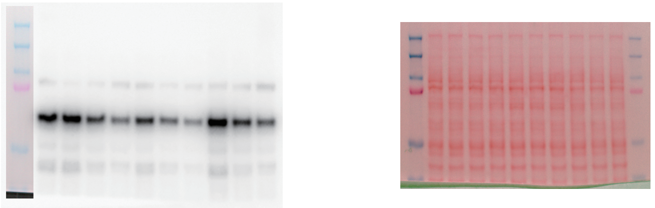

Fig. 6j c-MET Ponceau

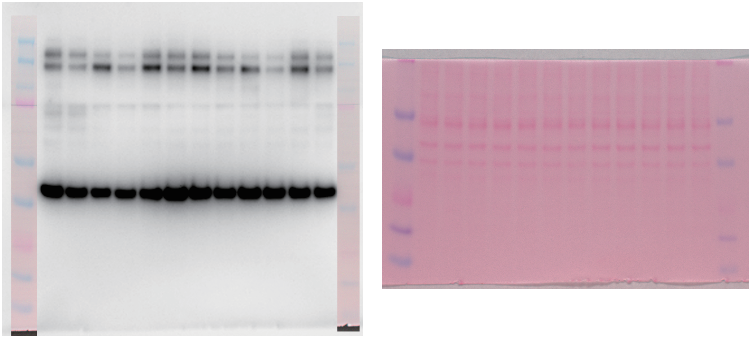

Fig.S1d

Replicate2

BRD4

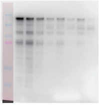

Ponceau

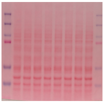

Replicate3

BRD4

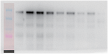

Ponceau

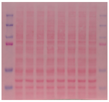

Fig.S1e

BRD4(JESS)

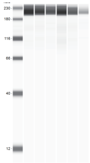

β-Actin(JESS)

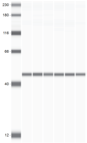

Fig.S2a

Replicate2

CUL2(IP)

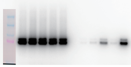

CUL2(input)

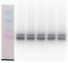

BRD4(input,IP)

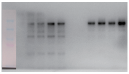

Replicate3

CUL2(IP)

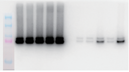

CUL2(input)

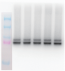

BRD4(input,IP)

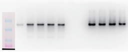

Fig.S2b

BRD4

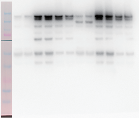

HSP90

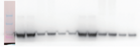

ETS1

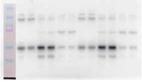

Ponceau(H3)

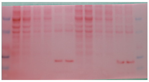

Fig.S2d

PSMD4(IP,input)

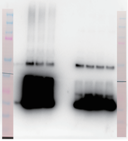

Rad23B(IP,input)

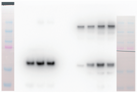

Ub(IP)

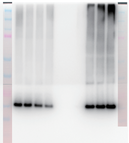

PSMB5(input,IP)

Ub(input)

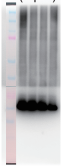

Fig.S3h

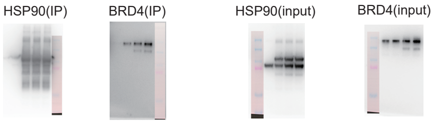

Supplement: Supplementary file 8 — Source data [file 41467_2024_49519_MOESM8_ESM.zip › Ohtake_SourceData_zip/Ohtake_SourceData_GelBlots.pdf]
